# Neuroprotective and Anti-Inflammatory Effects of Low–Moderate Dose Ionizing Radiation in Models of Alzheimer’s Disease

**DOI:** 10.3390/ijms21103678

**Published:** 2020-05-23

**Authors:** Sujin Kim, Yunkwon Nam, Chanyang Kim, Hyewon Lee, Seojin Hong, Hyeon Soo Kim, Soo Jung Shin, Yong Ho Park, Han Ngoc Mai, Sang-Muk Oh, Kyoung Soo Kim, Doo-Han Yoo, Weon Kuu Chung, Hyunju Chung, Minho Moon

**Affiliations:** 1 Department of Biochemistry, College of Medicine, Konyang University, Daejeon 35365, Korea; aktnfl3371@naver.com (S.K.); yunkwonnam@gmail.com (Y.N.); hsj122459@naver.com (S.H.); sooya1105@naver.com (H.S.K.); tlstnzz@konyang.ac.kr (S.J.S.); znf900809@naver.com (Y.H.P.); sangmuk_oh@konyang.ac.kr (S.-M.O.); 2 Department of Core Research Laboratory, Medical Science Research Institute, Kyung Hee University Hospital at Gangdong, Seoul 05278, Korea; praise1107@naver.com; 3 Department of Occupational Therapy, Konyang University, Daejeon 35365, Korea; lavine-woni@hanmail.net (H.L.); glovia@konyang.ac.kr (D.-H.Y.); 4 Department of Radiation Oncology, Kyung Hee University Hospital at Gangdong, Seoul 05278, Korea; maingochan244@gmail.com; 5 Department of Clinical Pharmacology and Therapeutics, Kyung Hee University School of Medicine, Seoul 02447, Korea; labrea46@naver.com

**Keywords:** Alzheimer’s disease, low-moderate dose ionizing radiation, radiotherapy, 5XFAD mice, neurodegeneration, neuroinflammation

## Abstract

Alzheimer’s disease (AD) is the most common cause of dementia. The neuropathological features of AD include amyloid-β (Aβ) deposition and hyperphosphorylated tau accumulation. Although several clinical trials have been conducted to identify a cure for AD, no effective drug or treatment has been identified thus far. Recently, the potential use of non-pharmacological interventions to prevent or treat AD has gained attention. Low-dose ionizing radiation (LDIR) is a non-pharmacological intervention which is currently being evaluated in clinical trials for AD patients. However, the mechanisms underlying the therapeutic effects of LDIR therapy have not yet been established. In this study, we examined the effect of LDIR on Aβ accumulation and Aβ-mediated pathology. To investigate the short-term effects of low–moderate dose ionizing radiation (LMDIR), a total of 9 Gy (1.8 Gy per fraction for five times) were radiated to 4-month-old 5XFAD mice, an Aβ-overexpressing transgenic mouse model of AD, and then sacrificed at 4 days after last exposure to LMDIR. Comparing sham-exposed and LMDIR-exposed 5XFAD mice indicated that short-term exposure to LMDIR did not affect Aβ accumulation in the brain, but significantly ameliorated synaptic degeneration, neuronal loss, and neuroinflammation in the hippocampal formation and cerebral cortex. In addition, a direct neuroprotective effect was confirmed in SH-SY5Y neuronal cells treated with Aβ_1–42_ (2 μM) after single irradiation (1 Gy). In BV-2 microglial cells exposed to Aβ and/or LMDIR, LMDIR therapy significantly inhibited the production of pro-inflammatory molecules and activation of the nuclear factor-kappa B (NF-κB) pathway. These results indicate that LMDIR directly ameliorated neurodegeneration and neuroinflammation in vivo and in vitro. Collectively, our findings suggest that the therapeutic benefits of LMDIR in AD may be mediated by its neuroprotective and anti-inflammatory effects.

## 1. Introduction

Alzheimer’s disease (AD), an age-related neurodegenerative disease, is the most common cause of dementia [1,2]. The main neuropathological hallmarks of AD are amyloid-β (Aβ) plaques precipitated by Aβ aggregation and neurofibrillary tangles composed of hyperphosphorylated tau proteins [3,4,5]. Other neuropathological features of AD include synaptic loss, neuronal death, and neuroinflammation in the brain [6,7]. The main symptoms of AD are cognitive impairment and memory loss, which accompany these neuropathological changes [6,8]. Although our understanding of AD pathology has significantly advanced, we have not yet identified effective therapeutic drugs or molecules for the treatment of AD [1]. Interestingly, it has been shown that non-pharmacological interventions, including low-dose ionizing radiation (LDIR), auditory stimulation, cognitive training and photic stimulation, can reduce neurodegeneration, Aβ accumulation and cognitive deficits in AD patients and experimental animal models [9,10,11,12,13]. Therefore, the idea that non-pharmacological interventions, in particular LDIR, could prevent or treat AD has gained attention [11,12,13,14].

In tracheobronchial amyloidosis patients, radiation therapy reduced amyloid-like deposits and improved symptoms [15,16]. Thus, radiation therapy is thought to be a promising therapeutic approach for the treatment of systemic amyloidosis. In addition, a number of studies have been conducted to investigate the efficacy of radiation therapy for the treatment of neurological disorders such as Parkinson’s disease, spinal cord injury, and AD [17,18,19]. Surprisingly, exposure to LDIR (0.32 Gy) significantly improved the symptoms of AD patients [13,19]. Furthermore, many studies showed that LDIR therapy significantly reduced AD-related pathology in animal models of AD [20,21,22]. However, the effects of LDIR on Aβ accumulation in AD animal models vary depending on the dose of radiation administered and the time point at which the irradiated animals are sacrificed [20,21,22,23,24,25]. Several previous reports, including our data, have demonstrated that LDIR (five fractions of 2 Gy) significantly reduced Aβ burden in an AD mouse model at 8 weeks post-irradiation [21,25,26]. By contrast, irradiation increased Aβ plaque in the brains of Aβ-overexpressing mice at 6 and 9 months post-irradiation [23,24]. Furthermore, a recent study revealed that although exposure to γ-irradiation did not affect levels of Aβ_42_ mRNA or protein, it had a neuroprotective effect in the brains of Drosophila AD models [20]. In another recent study, LDIR induced the upregulation of pre- and post-synaptic molecules in the brains of ApoE^−/−^Alzheimer’s mouse model [27,28]. Moreover, LDIR significantly reduced the levels of pro-inflammatory cytokines such as interferon gamma and tumor necrosis factor-alpha (TNF-α) in animal models of AD [21,27]. Taken together, several studies have shown that exposure of LDIR exhibited therapeutic effects on AD pathogenesis, but few studies have been conducted into the effect of LDIR therapy on neuronal death and neuroinflammation in vitro and in vivo.

Notably, some studies have examined the molecular changes to elucidate the early brain responses of exposure to LDIR in healthy animals [29,30,31]. Exposure of the mouse brain to LDIR induced the upregulation of genes for mitochondrial translocases and small heat shock proteins related with protective mechanisms at 30 min and 4 h post irradiation [30]. Moreover, LIDR-exposed healthy mice induced early transcriptional alterations in genes associated with AD [31]. Furthermore, in the brains of healthy mice, signaling pathways involved in axonal guidance signaling, integrin signaling, long-term depression, G-protein coupled receptor signaling, and long-term potentiation were found to be activated 4 h post irradiation [29]. Although the molecular changes induced by LDIR have been examined in normal/healthy brains [29,30], no studies have been conducted into the short-term effect of LDIR on AD-related pathology, including neuronal death, synaptic loss, neuroinflammation and Aβ-related pathology in animal models of AD.

In the present study, Aβ-overexpressing transgenic mice were exposed to low–moderate dose ionizing radiation (LMDIR) (9 Gy given in five fractions of 1.8 Gy) to investigate the short-term effect of LMDIR on Aβ-related pathology, neuronal/synaptic loss and neuroinflammation. Subsequently, we examined the in vitro effects of LMDIR on cell viability, pro-inflammatory cytokine production and nuclear factor-kappa B (NF-κB) pathway activation in Aβ-treated neuroblastoma SH-SY5Y cells and mouse microglial BV-2 cells.

## 2. Results

### 2.1. Short-Term Effect of LMDIR Treatment on Aβ Deposition in the Subiculum and Cerebral Cortex of Aβ-Overexpressing Transgenic Mice

**Figure 1:**
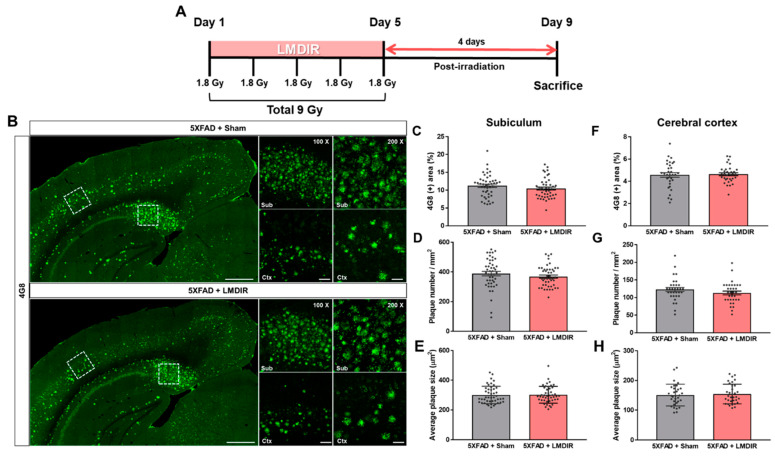
Effect of low–moderate dose ionizing radiation (LMDIR) on Aβ deposition in the subiculum and cerebral cortex of 5XFAD mice. (**A**) Schematic diagram of the experimental procedure. 5XFAD mice were exposed to sham or LMDIR (9 Gy in five fractions), and then sacrificed 4 days after the final irradiation. (**B**) Representative images of 4G8 immunoreactivity in the subiculum and cerebral cortex of sham- and LMDIR-exposed 5XFAD mice. LMDIR did not affect the (**C**) 4G8-positive area (%), (**D**) number of 4G8-positive plaques, or (**E**) average size of the 4G8-positive plaques in the subiculum. LMDIR did not affect the (**F**) 4G8-positive area (%), (**G**) number of 4G8-positive plaques, or (**H**) average size of the 4G8-positive plaques in the cerebral cortex. Data are presented as mean ± SEM (*n* = 7 in each group). Scale bar = 50 and 100 μm (Sub and Ctx) or 500 μm (hemisphere).

It has been shown that LDIR at a dose rate of 2 Gy/fraction for 5 days significantly reduced amyloid plaque deposition in the brains of Aβ-overexpressing transgenic mice at 2 months post irradiation [21]. To examine the short-term effect of LMDIR on Aβ accumulation in the brain, we estimated Aβ burden in the brain sections of sham- and LMDIR-exposed 5XFAD mice at 4 days post irradiation (Figure 1A and Supplementary Figure S1). Aβ plaques were stained with an anti-4G8 antibody, and analyzed by area fractions, number and average size in the subiculum and the cerebral cortex (Figure 1B–H). The depositions of amyloid plaques in radiation-exposed 5XFAD were not significantly different compared with sham-exposed 5XFAD mice at 4 days after LMDIR over 5 days. For the first time, we demonstrated that LMDIR had no significant short-term effect on Aβ pathology in the Aβ-overexpressing transgenic mice.

### 2.2. Inhibitory Effects of LMDIR on Synaptic and Neuronal Loss in the Hippocampal Formation of Aβ-Overexpressing Transgenic Mice

**Figure 2:**
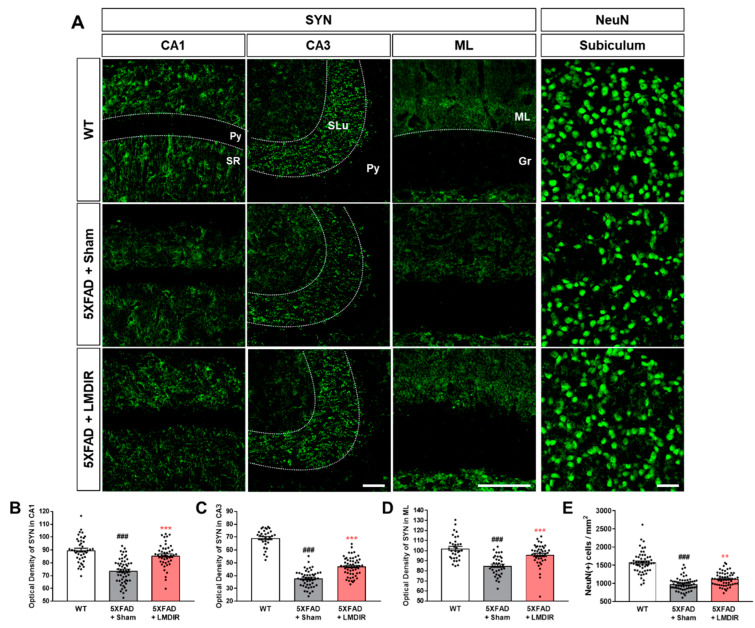
Protective effect of LMDIR against neurodegeneration in the hippocampal formation of 5XFAD mice. (**A**) Representative images of immunofluorescent staining of the hippocampal formation for NeuN, a marker of neurons, and SYN, a marker of pre-synaptic terminals. The fluorescence intensity of SYN immunoreactivity in the (**B**) CA1, (**C**) CA3 and (**D**) ML was significantly lower in the sham-exposed 5XFAD than in the WT mice. However, the decrease of SYN immunoreactivity in the brain of 5XFAD mice was significantly improved by LMDIR exposure. (**E**) The number of NeuN (+) cells per area was significantly lower in the sham-exposed 5XFAD mice than in the WT mice. However, the reduction of NeuN (+) cells in the brains of 5XFAD mice was significantly improved by LMDIR exposure. Data are presented as mean ± SEM (*n* = 7 in each group). ^###^ *p* < 0.001: WT mice versus sham-exposed 5XFAD mice. ** p < 0.01 and *** *p* < 0.001: sham-exposed 5XFAD mice versus LMDIR-exposed 5XFAD mice. Scale bar = 40 μm for CA1, CA3 and subiculum. Scale bar = 100 μm for ML. Py, pyramidal tract; SR, stratum radiatum; SLu, stratum lucidum; ML, molecular layer; Gr, granular layer; LMDIR, Low–moderate dose ionizing radiation; NeuN, neuronal nuclear antigen; SYN, synaptophysin.

The loss of synapses and neurons is a major pathological feature of AD which is known to cause memory loss [8]. Although LDIR has been shown to reduce Aβ_42_-induced neuronal death in Drosophila models of AD [20], no study has investigated the neuroprotective or synapto-protective effects of LDIR in Aβ-overexpressing rodent models. Therefore, we examined the expression of synaptic and neuronal marker proteins in the brains of 4-month-old 5XFAD mice, which exhibit no cognitive deficits [32]. To visualize axon terminals and neuronal cells, we immunohistochemically stained brain sections from the animals with synaptophysin (SYN) and neuronal nuclear antigen (NeuN), respectively (Figure 2A). The histological analysis revealed that 5XFAD mice showed a decrease of both SYN immunoreactivity in the hippocampus (Figure 2B–D) and fewer NeuN-positive cells in the subiculum (Figure 2E) of 5XFAD mice, compared with wild-type mice. However, the LMDIR-treated 5XFAD mice exhibited significant increase in SYN immunoreactivity and NeuN positive cells compared with sham-treated 5XFAD mice (Figure 2). These findings suggest that exposure to LMDIR significantly reduced synaptic and neuronal loss in brains with Aβ.

### 2.3. Attenuating Effects of LMDIR on Neuroinflammation in the Subiculum and Cerebral Cortex of 5XFAD Mice

**Figure 3:**
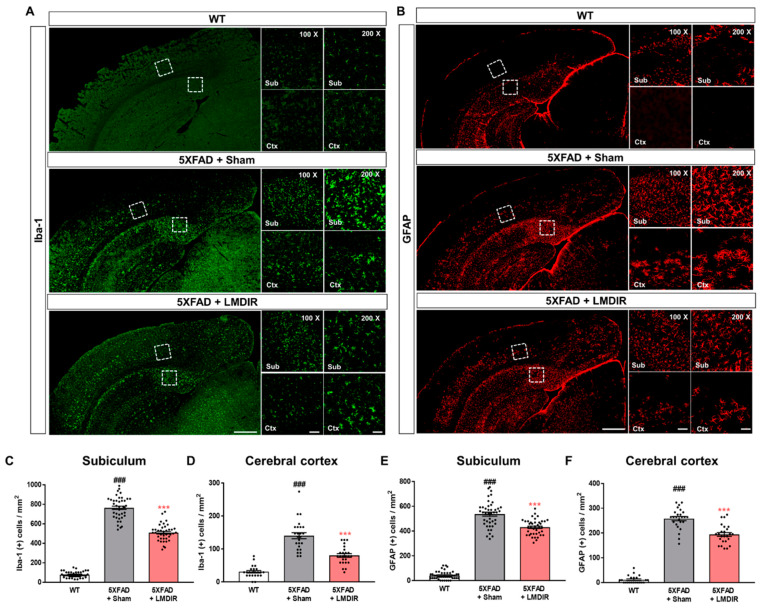
Inhibitory effect of LMDIR on neuroinflammation in the subiculum and cerebral cortex of 5XFAD mice. (**A**) Representative images of immunofluorescent staining of the subiculum and cerebral cortex for Iba-1, a marker of microglia. (**B**) Representative images of immunofluorescent staining of the subiculum and cerebral cortex for GFAP, a marker of astrocytes. (**C**,**D**) The number of Iba-1 (+) cells was significantly higher in the subiculum and cerebral cortex of 5XFAD mice than that of the WT mice. In contrast, the number of Iba-1 (+) cells was significantly lower in the subiculum and cerebral cortex of LMDIR-exposed 5XFAD mice than that of the sham-exposed 5XFAD mice. (**E**,**F**) The number of GFAP (+) cells was significantly higher in the subiculum and cerebral cortex of 5XFAD mice than the WT mice. However, the number of GFAP (+) cells was significantly lower in the subiculum and cerebral cortex of LMDIR-exposed 5XFAD mice than that of the sham-exposed 5XFAD mice. Data are presented as mean ± SEM (*n* = 7 in each group). ^###^ *p* < 0.001: WT mice versus sham-exposed 5XFAD mice. *** *p* < 0.001: sham-exposed 5XFAD mice versus LMDIR-exposed 5XFAD mice. Scale bar = 50 and 100 μm (Sub and Ctx) or 500 μm (hemisphere). LMDIR, low–moderate dose ionizing radiation; Iba-1, ionized calcium-binding adaptor molecule 1; GFAP, glial fibrillary acidic protein.

Chronic accumulation of Aβ in the brain induces neuroinflammation, which is characterized by the activation of microglia and astrocytes [33]. In addition, glial activation can promote the release of pro-inflammatory cytokines, which further aggravates Aβ-induced neurodegeneration [34,35]. To examine the effect of LDIR on glial responses in the AD brain, we performed immunohistochemical staining for microglial and astrocytic markers in the brains of 5XFAD mice. Ionized calcium-binding adaptor molecule 1 (Iba-1) and glial fibrillary acidic protein (GFAP) were employed as markers of microglia and astrocytes, respectively (Figure 3A,B). The number of Iba-1- and GFAP-positive cells in the subiculum and cerebral cortex was significantly higher in the 5XFAD mice than in the wild-type mice (Figure 3C–F). In contrast, the number of microglia and astrocytes was significantly lower in the LMDIR-exposed 5XFAD mice than that in the sham-exposed 5XFAD mice (Figure 3C–F). In addition, we investigated the microgliosis in the subgranular zone (SGZ) and subventricular zone (SVZ) (Supplementary Figure S2). We confirmed that LMDIR-exposed 5XFAD mice exhibited a significantly lower number of Iba-1-positive cells in both SGZ and SVZ than that of sham-exposed 5XFAD mice. Taken together, these results demonstrated that short-term LMDIR exposure significantly reduced both microgliosis and astrogliosis in the brains of animal models of AD.

**Figure 4:**
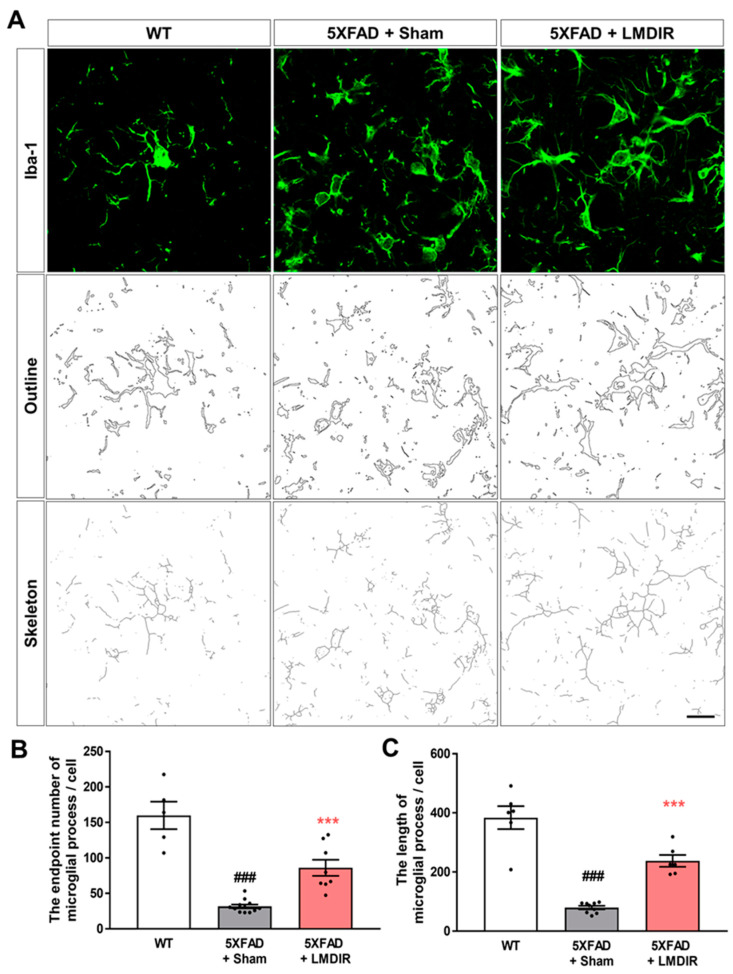
The effect of LMDIR on transformation from amoeboid to ramified microglial morphology changes in the subiculum of 5XFAD mice. (**A**) Representative images of immunofluorescent staining, cell outlines and skeleton structure of the subiculum for Iba-1, a marker of microglia. (**B**) The number of microglial process endpoints was significantly lower in the subiculum of 5XFAD mice than that of the WT mice. In contrast, the number of microglial process endpoints was significantly higher in the subiculum of LMDIR-exposed 5XFAD mice than that of the sham-exposed 5XFAD mice. (**C**) Microglial process length was significantly shorter in the subiculum of 5XFAD mice than that of the WT mice. However, microglial process length was significantly longer in the subiculum of LMDIR-exposed 5XFAD mice than that of the sham-exposed 5XFAD mice. Data are presented as mean ± SEM (*n* = 7 in each group). ^###^ *p* < 0.001: WT mice versus sham-exposed 5XFAD mice. *** *p* < 0.001: sham-exposed 5XFAD mice versus LMDIR-exposed 5XFAD mice. Scale bar = 20 μm. LMDIR, low–moderate dose ionizing radiation; Iba-1, ionized calcium-binding adaptor molecule 1.

Upon microglial activation by AD pathogenesis, infection or any other neurodegenerative stimulus, microglial cells retract their ramification and transform into amoeboid or spherical shape [36]. Activated microglia produce pro-inflammatory cytokines and lead neurotoxicity [37,38]. To confirm that microglial morphology changes after LMDIR in AD, we analyzed the processes of Iba-1-positive cells in the 5XFAD mouse brain using the ImageJ program. We found microglial morphology changes though quantitative outcome of both skeleton and fractal analysis (Figure 4). The number of microglial process endpoints and the length of process in the subiculum was significantly lower in the 5XFAD mice than that in the wild-type mice (Figure 4B,C). In contrast, the number of microglial process endpoints and length of process in the subiculum was significantly higher in the radiation-exposed 5XFAD mice than that in the sham-exposed 5XFAD mice. These findings suggest that exposure to LMDIR significantly affects change of microglial morphology in brains with Aβ.

### 2.4. Direct Effects of LMDIR on Aβ-Induced Cell Death in Cultured Neurons

**Figure 5:**
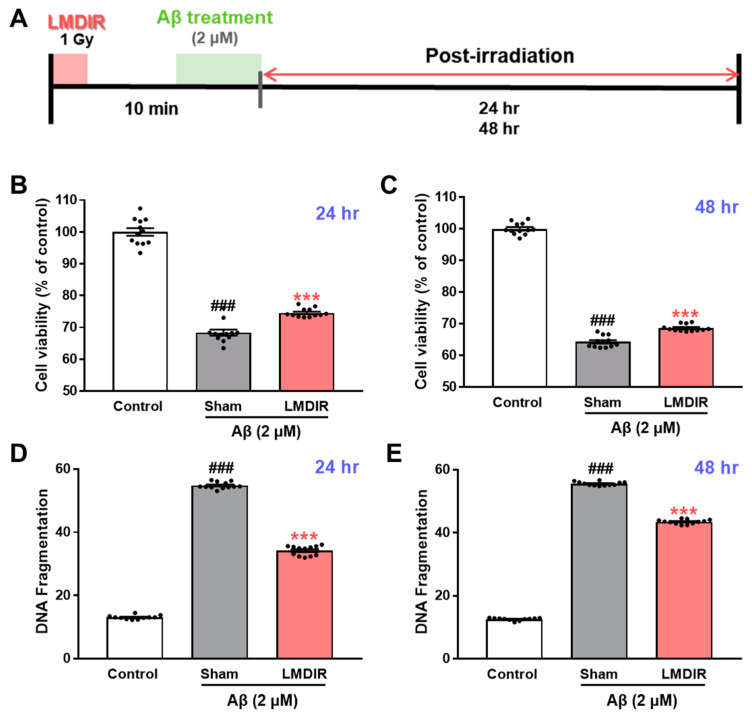
Direct neuroprotective effect of LMDIR against Aβ toxicity in cultured neuronal cells. (**A**) Outline of the experimental design. SH-SY5Y cells were exposed to LDIR (1 Gy), and then incubated with 2 μM Aβ for 24 or 48 h. (**B**,**C**) Cell viability was measured using an MTT assay. The cell viability of the LMDIR- and Aβ-treated SH-SY5Y cells was significantly higher than that of the sham- and Aβ-treated SH-SY5Y cells 24 and 48 h after irradiation. (**D**,**E**) Cell death was evaluated by measuring DNA fragmentation using an ELISA kit. The DNA fragmentation in LMDIR- and Aβ-treated SH-SY5Y cells was significantly lower than that of sham- and Aβ-treated SH-SY5Y cells 24 h and 48 h after irradiation. Data are presented as mean ± SEM. ^###^ *p* < 0.001: control versus sham-exposed SH-SY5Y cells. *** *p* < 0.001: sham-exposed SH-SY5Y cells versus LMDIR-exposed SH-SY5Y cells. LMDIR, low–moderate dose ionizing radiation; Aβ, amyloid-β.

To study the direct effect of LMDIR on neuronal death in vitro, we determined whether LDIR can also protect cultured neurons against Aβ toxicity using 3-(4,5-dimethylthiazol-2-yl)-2,5-diphenyltetrazolium bromide (MTT) assay and DNA fragmentation assay. SH-SY5Y cells were exposed to 1 Gy of radiation and treated with Aβ (Figure 5A). LMDIR treatment significantly ameliorated Aβ-induced neuronal loss, compared with sham- and Aβ-treated cells 24 h or 48 h after irradiation (Figure 5B,C). In addition, the sham- and Aβ-treated SH-SY5Y cells exhibited a greater amount of fragmented DNA than the control cells. However, LMDIR- and Aβ-treated cells exhibited the reduction of fragmented neuronal DNA 24 and 48 h after irradiation (Figure 5D,E). These results suggest that LMDIR can directly alleviate Aβ-induced neuronal loss.

### 2.5. Alleviatory Effect of LMDIR on Aβ-Induced Neuroinflammatory Cytokine Production and NF-κB Pathway Activation in BV-2 Cells

**Figure 6:**
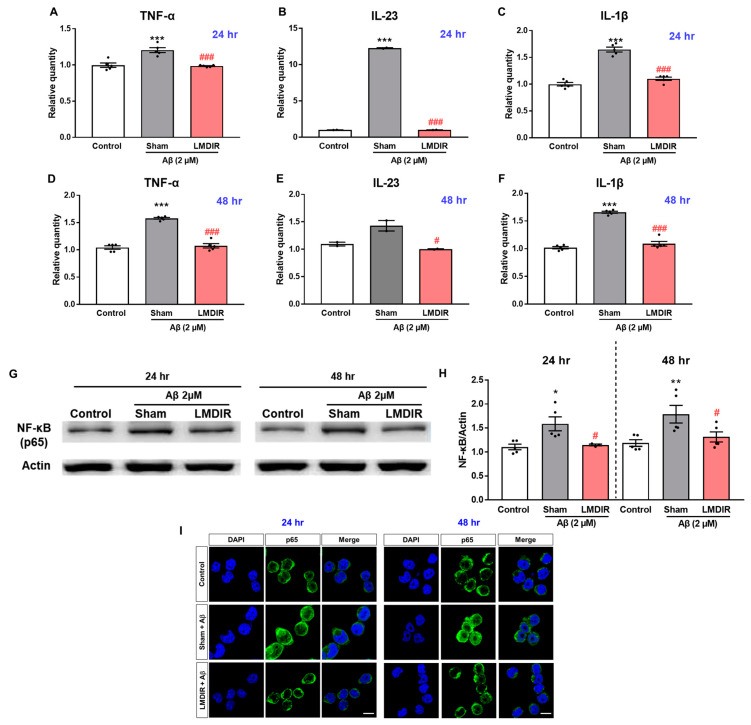
Anti-neuroinflammatory effect of LMDIR against Aβ toxicity in cultured microglia cells. BV-2 cells were exposed to LMDIR (1 Gy), and then incubated with 2 μM Aβ for 24 or 48 h. (**A**–**F**) The mRNA levels of pro-inflammatory cytokines in BV-2 cells 24 and 48 h after treatment with Aβ or/and LMDIR were measured using quantitative real-time PCR. LMDIR exposure ameliorated the Aβ-induced production of pro-inflammatory cytokines in BV-2 cells. (G,H) Representative Western blots. For NF-κB detection, Western blotting was performed using p65 and actin antibodies in the lysate of BV-2 cells. p65 were normalized to actin. (**I**) Cellular localization and expression of NF-κB p65 were analyzed by immunofluorescence staining. After Aβ treatment, the translocation of NF-κB p65 into the nucleus was remarkably increased in sham-treated microglia cells compared to control groups. However, the Aβ-induced translocation was inhibited in LMDIR- and Aβ-treated microglia cells compared to sham- and Aβ-treated microglia cells. Scale bar = 10 μm. Data are presented as mean ± SEM. ^#^ *p* < 0.05 and ^###^ *p* < 0.001: control versus sham-exposed BV-2 cells. * p < 0.05, ** *p* < 0.01 and *** *p* < 0.001: sham-exposed BV-2 cells versus LMDIR-exposed BV-2 cells. LMDIR, low–moderate dose ionizing radiation; Aβ, amyloid-β, NF-κB, nuclear factor-kappa B; TNF-α, tumor necrosis factor-alpha; IL, interleukin.

In a previous study, LDIR reduced the levels of pro-inflammatory cytokines, particularly TNF-α, in an AD animal model [27]. To further examine the inhibitory effect of LDIR on the production of pro-inflammatory molecules by Aβ-activated microglia, we measured the mRNA levels of pro-inflammatory cytokines in Aβ- or/and LMDIR-treated microglial cells. Sham-irradiated BV-2 cells treated with Aβ for 24 or 48 h exhibited increased TNF-α, IL-23 and IL-1β levels compared to control cells not treated with Aβ (Figure 6A–F). In contrast, LMDIR significantly inhibited the transcription of pro-inflammatory cytokines in Aβ-treated BV-2 cells (Figure 6A–F). For the first time, we demonstrated that LMDIR therapy has a direct anti-inflammatory effect on the Aβ-treated mouse microglia BV-2 cell line. Interestingly, it has been widely known that pro-inflammatory cytokine production is dependent on the NF-κB pathway [39]. In order to identify the signaling pathways involved in the anti-inflammatory effect of LMDIR in Aβ-treated microglia, we investigated the expression of p65 NF-κB using Western blot analysis. LMDIR inhibited the Aβ-induced upregulation of p65 NF-κB in BV-2 cells, compared with the sham- and Aβ-treated BV-2 cells (Figure 6G,H). Moreover, LMDIR markedly reduced the Aβ-mediated increase of expression and nuclear translocation of NF-κB p65 compared with the sham- and Aβ-treated BV-2 cells (Figure 6I). These results suggest that LMDIR may inhibit the Aβ-induced production of neuroinflammatory cytokines by microglia by suppressing NF-κB pathways.

## 3. Discussion

Given that almost all clinical trials of AD candidate drugs performed thus far have failed, the idea that non-pharmacological approaches such as radiation therapy could be used to treat AD is gaining interest [9,11,12]. Although the number of experimental and clinical studies into the effect of low-dose radiotherapy in AD is rapidly increasing, few studies have investigated the mechanisms underlying its therapeutic effects. Moreover, there has been no evidence showing the short-term and direct effects of LMDIR on Aβ pathology, neurodegeneration and neuroinflammation during AD pathogenesis in Aβ-overexpressing transgenic animals. In this study, we demonstrated that LMDIR inhibited neurodegeneration and neuroinflammation in Aβ-overexpressing mice (Figure 2, Figure 3 and Figure 4). Furthermore, we found that LMDIR directly suppressed Aβ-induced neuronal death and neuroinflammatory responses in a neuroblastoma SH-SY5Y cell line and a mouse microglia BV-2 cell line (Figure 5 and Figure 6). Specifically, LMDIR suppressed the upregulation and nuclear translocation of NF-κB p65 in Aβ-treated microglia (Figure 6G–I). Thus, we suggested that LDIR therapy may be a promising and effective approach for the treatment of AD through its effects on neuroprotective and anti-inflammatory effects. However, our study cannot exclude harmful effects such as radiation-induced neuronal dysfunction and the alteration of gene expression associated with neurotoxicity.

**Figure 7:**
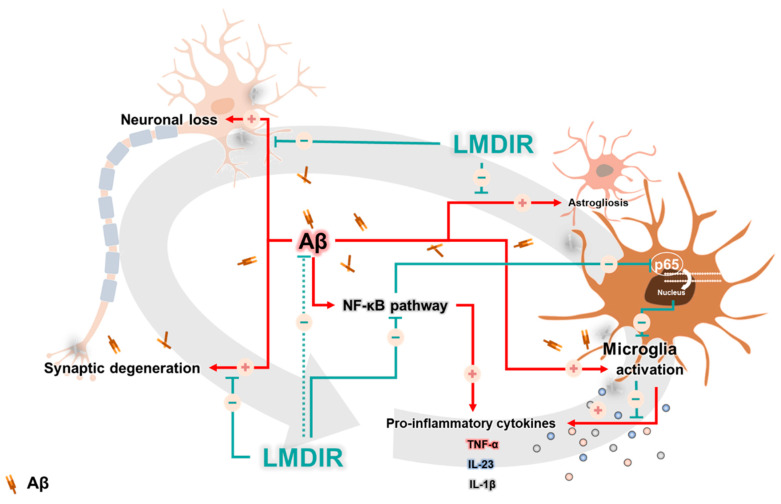
A possible mechanism underlying the therapeutic benefits of LMDIR treatment in AD. In the Aβ-overexpressing brains, a vicious cycle between neuronal loss and glial activation is established and promotes the onset of AD. Aβ induces neuronal loss and synaptic degeneration, and the degenerating neurons then release microglial activators, resulting in the activation of glial cells. In addition, activated microglial cells produce neurotoxic cytokines. Interestingly, the NF-κB signaling pathway is associated with upregulation of the pro-inflammatory cytokine from glial cells. LMDIR treatment inhibits neurodegeneration and neuroinflammation by inhibiting the NF-κB signaling pathway. Stimulatory pathways are indicated by + arrows and − arrows indicate inhibitory pathways. Low–moderate dose ionizing radiation (LMDIR); AD, Alzheimer’s disease; Aβ, amyloid-β; NF-κB, nuclear factor kappa-B.

In AD, neuronal loss and synaptic generation are caused by both Aβ and Aβ-mediated neuroinflammation [40,41]. Microglia activated by Aβ are able to produce neurotoxic pro-inflammatory cytokines such as IL-23, IL-1β and TNF-α [35]. In addition, the pro-inflammatory cytokine TNF-α is thought to be involved in the pathophysiology of AD by promoting abnormal Aβ production, synaptic degeneration and neuronal death [42]. Moreover, degenerating neurons may also directly activate microglia by secreting microglial activators such as laminin and matrix metalloproteinase-3 (MMP-3) [43]. This self-perpetuating cycle between degenerating neurons and activated microglia promotes the progression of AD (Figure 7). Furthermore, this continuous and vicious cycle can also contribute to cognitive impairment, a symptom of AD. Thus, an intervention that promotes the destruction of this vicious cycle may effectively inhibit the progression of AD. Surprisingly, previous studies have shown that LDIR strongly suppressed neuronal death in an Aβ-overexpressing Drosophila model [20] and reduced microglial activation in the ApoE^−/−^AD mouse model [27]. Consistent with previous studies, we demonstrated that LMDIR significantly ameliorated neuronal death and neuroinflammation both in vitro and in vivo (Figure 2, Figure 3, Figure 4, Figure 5 and Figure 6). Notably, for the first time, we demonstrated that LMDIR inhibited synaptic loss, astrogliosis and NF-κB pathway activation in models of AD. To the best of our knowledge, our study is the first to demonstrate the neuroprotective and anti-inflammatory effects of LMDIR, suggesting that LMDIR can inhibit the vicious cycle which is established between degenerating neurons and activated microglia in AD. Nonetheless, our in vitro and in vivo results regarding the effect of LMDIR on neuroprotective and anti-inflammatory effect should be interpreted with caution.

The aim of the present study was to examine the short-term effects of LMDIR exposure on AD-related pathology. LMDIR exposure did not affect Aβ deposition in the subiculum and cerebral cortex of 5XFAD mice at 4 days post irradiation (Figure 1). The relationship between radiation therapy and Aβ pathology remains controversial. Some studies have shown that LDIR does not affect or promote Aβ deposition in animal models of AD [20,23,24]. On the other hand, other studies have revealed a reduction of amyloid plaque and plaque size in the brains of LDIR-treated AD animal models 2 months after irradiation [21,22,25]. Although the mechanism by which LDIR reduces Aβ deposition has not been fully determined [44], it seems likely that Aβ clearance after radiation therapy may be mediated by neuroinflammatory responses such as Aβ phagocytosis. One previous study proved that upregulated inflammatory cytokine production and microglial activation precedes Aβ deposition in an AD animal model [45]. Furthermore, inhibition of the neuronal death may contribute to the alleviation of a pro-inflammatory cytokine, which is mediated by suppression of microglia activation through the prohibition of microglia activators such as laminin and MMP-3, released from dead or stressed neurons [43]. Based on our results and those of previous studies, the LMDIR-mediated reduction of Aβ plaques may be preceded by the inhibition of neuronal loss and alleviation of neuroinflammatory responses. Nevertheless, our knowledge of the effects of LDIR on Aβ is limited [44], and the mechanisms underlying the therapeutic benefits of LDIR are not fully understood. Therefore, in a future study, we aim to investigate the direct effect of LDIR on Aβ aggregation or dissociation and the protective effect of radiation on the high concentration of Aβ_1–42_. In addition, the animal model used in this study is an Aβ-overexpressing transgenic mouse. Thus, we could not examine the effects of LDIR on other causative factors for AD, such as tau. In future studies, we aim to investigate the effect of LDIR on tau pathology in vitro and in vivo.

Surprisingly, in the present study, upregulation of TNF-α, IL-23, IL-1β and NF-κB was significantly decreased after LMDIR irradiation in the brain with AD (Figure 6). Consistent with our findings, in previous studies, LDIR reduced the levels of pro-inflammatory cytokines, particularly TNF-α, in AD animal models [24,27]. TNF-α is overproduced in neurodegenerative conditions and has been identified as one of the main causes accelerating and sustaining progression of AD pathology [46,47]. In the AD brain, TNF-α promotes chronic neuroinflammation by inducing differentiation of microglial cells into M1 phenotypes and reduces the phagocytosis of Aβ by microglial cells [35,48]. Meanwhile, the transcription factor NF-κB is a crucial up-regulator [49] that increases the production of TNF-α, and abnormally high levels of this factor have been found in AD patients [50,51]. Interestingly, it has been reported that TNF-α might increase Aβ production by directly acting on BACE1 [51,52]. In addition, the phagocytosis of Aβ by microglial cells is dependent on the NF-κB pathway [53]. Moreover, NF-κB-induced TNF-α overproduction can cause neurodegeneration through interaction with Fas ligand [54,55,56]. IL-23 and IL-1β are other pro-inflammatory cytokines produced via the NF-κB pathway [49,57]. IL-23 is known to play an important role in promoting the immune response [58]. In addition, IL-1β has been reported to upregulate Aβ production and act as a major pro-inflammatory cytokine in the AD brain [35,59]. Thus, regulating IL-1β could be effective in alleviating neuroinflammation of AD. Interestingly, NF-κB-specific target genes contribute to the generation of reactive oxygen intermediates, which may directly cause neurotoxicity [60]. Therefore, the NF-κB pathway can be an effective therapeutic target for neuroinflammation in AD [46,61]. Taken together, the therapeutic effect of LMDIR in AD may be mediated via the inhibition of NF-κB signaling (Figure 7). Furthermore, for the first time, we demonstrated the molecular mechanism of anti-inflammatory action of LMDIR in microglial cells under AD conditions.

It has been suggested that possible therapeutic effects of LDIR on AD might be mediated by regeneration of the myelin sheath, inhibition of neurodegeneration by oxidative stress and increase of adult neurogenesis [62]. In the Drosophila model of AD, the neuroprotective effect of LDIR was mediated through activation of AKT signaling and inhibition of the p38 pathway [20]. Exposure to LDIR upregulates the expression of genes involved in protective mechanisms such as cell cycle control and DNA synthesis and repair in the brains of healthy mice [30]. In addition, LDIR exposure induces genes associated with platelet-derived growth factor signaling, one of numerous growth factors, in the brains of healthy mice [29]. Further, radiation-treated AD mice showed significantly reduced latency in finding the platform in a Morris water maze compared to sham-treated AD mice [21]. Another previous study demonstrated that male LDIR-treated AD mice exposed to 0.5 Gy exhibited a reduction in fear memory in a contextual fear conditioning test [22]. Thus, behavioral tests for various types of memory might be advantageous for evaluating the neuroprotective effects of LDIR in AD. Further studies are needed to elucidate the detailed molecular mechanisms underlying the neuroprotective effect of LDIR in the brains of Aβ-overexpressing rodents and to demonstrate the neuroprotective and anti-inflammatory effect of LDIR in the various transgenic mouse model for AD.

Surprisingly, low-dose radiation therapy in the form of whole-body computed tomography (CT) scans improved the symptoms of a patient with AD. The patient was exposed to a total radiation dose of around 0.32 Gy, which improved her cognition, memory, movement, appetite and speech [13,19]. Based on this case report, six clinical studies into the use of LDIR for the treatment of AD are currently ongoing according to clinicaltrials.gov (ClinicalTrials.gov Identifier: NCT02769000; NCT04203121; NCT03352258; NCT02359864; NCT03597360; and NCT00599469), one of which is being conducted by our research team (NCT04203121). In these studies, AD progression is being evaluated through neurocognitive tests and amyloid positron emission tomography scans. While the number of studies investigating the use of LDIR for the treatment of AD is increasing, more studies are needed to illuminate the molecular mechanisms underlying the effects of LDIR. Our results revealed that LDIR treatment significantly ameliorated Aβ-related neurodegeneration and neuroinflammation (Figure 7).

## 4. Materials and Methods

### 4.1. Animals

5XFAD mice express five familial AD-related mutations in the human PSEN1 gene (M146 and L286) and APP gene (K670N/M671L, V717I, and I716V). We purchased 5XFAD mice from the Jackson Laboratory (Bar Harbor, ME, USA). The transgenic mice were genotyped using polymerase chain reaction (PCR) and their wild-type littermates were used as a control group. The animals were maintained in accordance with the Guide for the Care and Use of Laboratory Animals (National Institutes of Health publication No. 85–23, revised 1985) and the Animal Care and Use Guidelines of Kyung Hee University Hospital at Gangdong. This animal research was approved by the ethics committee of Kyung Hee University Hospital at Gangdong (project identification code: KHNMC AP 2017-003, date: 7 August 2017). A total of 21 mice were used in this study and divided into three groups of seven mice: (1) wild-type mice, (2) sham-exposed 5XFAD mice and (3) LMDIR-exposed 5XFAD mice. Four-month-old female mice were used for all animal experiments.

### 4.2. Cranial Irradiation Procedure

For X-ray irradiation, Zoletil (2.5 mg/kg)-anesthetized mice were placed on an immobilizer. An X-ray irradiation system (LEP-300, Auracare^®^, Gyeonggi-do, Korea) and X-ray tube (320 kVp, 15 mA, Varian Medical Systems, Inc., Palo Alto, CA, USA) were used in the head irradiation at a total dose of 9 Gy (1.8 Gy per fraction) for five consecutive days (Figure 1A). The dose of LMDIR in this experiment was slightly modified from a previous study [21].

### 4.3. Preparation of Brain Tissue

The animals were sacrificed 4 days after the final LMDIR treatment. Anesthetized mice were subjected to transcardial perfusion fixation with ice-cold 4% paraformaldehyde (PFA) in phosphate-buffered saline (PBS). The brains were extracted and post fixed in 4% PFA for 20 h at 4 °C and immersed in 30% sucrose solution at 4 °C for 3 days for cryoprotection. The brains were cut into 30 μm thick sections with a CM1850 cryostat microtome (Leica Biosystems, Wetzlar, Germany). The brain tissue sections were stored in a cryoprotectant solution (25% ethylene glycol and 25% glycerol in 0.05 M phosphate buffer) at 4 °C until use.

### 4.4. Immunofluorescence Labeling

For immunofluorescent labeling, four to five coronal sections were obtained at the level of the SVZ (1.18 and 0.02 mm from the bregma) and hippocampal formation (−1.58 and −3.80 mm from the bregma) based on Paxinos and Franklin’s “The Mouse Brain in Stereotaxic Coordinates”(Supplementary Figure S3) [63]. The brain tissues were washed in PBS using the free-floating method on a Titramax 101 orbital shaker (Heidolph, Schwabach, Germany). The sections were incubated overnight at 4 °C with the following primary antibodies: mouse anti-4G8 antibody (1:2,000; Cat.# 800701, BioLegend, San Diego, CA, USA), mouse anti-NeuN antibody (1:100; Cat.# MAB377, Merck KGaA, Darmstadt, Germany), mouse anti-SYN antibody (1:500; Cat.# S5768, Sigma-Aldrich, St. Louis, MO, USA), goat anti-Iba-1 antibody (1:500; Cat.# ab5076, Abcam, Cambridge, UK), or rat anti-GFAP antibody (1:1000; Cat.# 13-0300, Thermo Fisher Scientific Inc., Waltham, MA, USA). They were incubated individually in PBS containing 0.3% Triton X-100 and 0.5 mg/mL bovine serum albumin (BSA). Before they were incubated with the anti-4G8 antibody, the brain sections were incubated with 70% formic acid for 20 min for antigen retrieval. After they were washed in PBS three times for 5 min each, the sections were incubated with a secondary antibody for 50 min at room temperature. The following secondary antibodies were used in this study: donkey Alexa 488-conjugated anti-mouse IgG (1:200; Cat.# A21202, Thermo Fisher Scientific Inc.), donkey Alexa 488-conjugated anti-goat IgG (1:200; Cat.# A11055, Thermo Fisher Scientific Inc.), and donkey Alexa 594-conjugated anti-rat IgG (1:200; Cat.# A21209, Thermo Fisher Scientific Inc.). All antibodies were diluted in PBS containing 0.3% Triton X-100. The sections were then washed in PBS, mounted on glass slides, and cover slipped with Fluoroshield™ with DAPI (Sigma-Aldrich) to counterstain the nuclei.

### 4.5. Image Acquisition and Analysis

All fluorescence images were captured with a Zeiss LSM 700 confocal microscope (Carl Zeiss AG, Oberkochen, Germany) and analyzed using ImageJ software (National Institutes of Health, Bethesda, MD, USA). To quantify the number of 4G8 (+), NeuN (+), Iba-1 (+) and GFAP (+) cells per mm^2^ and mm, 28 to 35 images of the immunostained brain sections were quantified as follows: (1) the positively-stained areas were manually outlined using the paintbrush tool; (2) the images were changed to 8-bit images; (3) the images were thresholded to eliminate background signals; (4) the anatomical region of the hippocampal formation, cerebral cortex and subventricular zone was defined based on DAPI; (5) the thresholded images of the defined brain region were quantified using the “analyze particles” tool and the “count” value was read; and (6) the “count” value was divided by the area of the defined region. The analysis methods for the average size of 4G8 (+) plaques are equal to steps 2–4 regarding the methods for quantifying the number of immunostained cells per mm^2^. Steps five and six differ as follows: for step five, the thresholded images of the defined brain region were quantified by the “analyze particles” tool for the “average size” measurement of the positive signals; for step six, the images were quantified using “average size” measurement. The quantification methods of percentage of 4G8 (+) area are also equal to steps 2–4 regarding the methods for quantifying the number of immunostained cells per mm^2^. Steps five and six are different as follows: in step five, the thresholded images of the defined brain region were quantified by the “analyze particles” tool for the “% area” measurement of the 4G8 (+) labeled signals; step six quantified using “% area” measurement. The methods of measurement of the optical density of SYN immunoreactivity were as follows: (1) manually drawing the region of interest (ROI); (2) selecting “analyze” and “measure” to give a numerical measurement to the fluorescence of the ROI.

### 4.6. Skeleton and Fractal Analysis

The ImageJ software and appropriate plugins were used prior to converting all images to skeletonized and outline images. The skeleton and fractal analysis was performed with reference to Young’s study [64]. Z-stacks were imported into the software and the slices containing individual Iba-1 positive cells were identified by manually scrolling through the Z-stack. Creating skeletonized images was done as follows: (1) cell somas were manually counted for each immunofluorescence image; (2) the images were changed to 8-bit images; (3) the images were adjust to brightness/contrast; (4) the images were applied to unsharp mask; (5) the images were converted to binary using threshold; (6) the images were applied to despeckle, close, and remove outliers functions; (7) the images were changed to skeletonize; (8) the skeletonized images were underwent for a skeleton analysis; (9) the “result” and “branch information outputs” from the skeletonized images were transported to an excel sheet; (10) raw data output from the skeleton analysis was sorted to “endpoint voxels” and “maximum branch length” from largest to smallest; (11) branch information output data were sorted to branch length from largest to smallest; (12) the raw data was removed to less than cutoff value (0.5 μm); and (13) the data was summed “endpoint voxels” and “branch length”. We summarized the number of microglial process endpoints and microglial process length from the Analyze Skeleton plugin data output and normalized all data by the number of microglia soma in individual images to calculate the number of microglia process endpoints/cell and microglia process length/cell. We extended our computer-aided morphologic analysis to include fractal analysis. To visualize fractal images we did the following: (1) cell somas were manually counted for in each immunofluorescence image; (2) the images were changed to 8-bit images; (3) the images were adjusted to brightness/contrast; (4) the images were applied to unsharp mask; (5) the images were converted to binary using threshold; (6) the images were applied to despeckle, close, and remove outliers function; and (7) images were changed to outlines.

### 4.7. Cell Culture

Human neuroblastoma SH-SY5Y cells and murine BV-2 microglial cells were maintained in Gibco Dulbecco’s Modified Eagle Medium: Nutrient Mixture F-12 containing 10% fetal bovine serum and 1% antibiotic-antimycotic in a 5% CO_2_ humidified incubator at 37 °C.

### 4.8. Measurement of Cell Viability and DNA Fragmentation

An MTT assay was performed to measure cell viability. SH-SY5Y cells were seeded in 96-well plates at a density of 10^5^ cells/mL. Immediately before radiation exposure, the culture dish was filled with media and all air gaps were eliminated. The cells were exposed to a single 1 Gy dose of LMDIR. After irradiation, the cells were treated with Aβ_1–42_ (2 μM) for 24 or 48 h. Then, MTT solution (Sigma-Aldrich) was added to each well, and the plates were incubated at 37 °C for 4 h. Following this, the MTT solution was removed and dimethyl sulfoxide was added. The absorbance was measured at 540 nm using a microplate reader (Molecular Devices LLC., CA, USA). DNA fragmentation was assessed using a Cell Death Detection ELISA kit (Cat.# 11544675001, Roche, Basel, Switzerland), according to the manufacturer’s instructions.

### 4.9. Determination of Pro-Inflammatory Cytokines

BV-2 cells were stimulated with Aβ_1–42_ (2 μM) and/or exposed to LMDIR for 24 or 48 h. Total mRNA was extracted from BV-2 cells using the Qiagen RNeasy Mini Kit (Qiagen, Hilden, Germany), according to the manufacturer’s instructions, and the mRNA was then transcribed to cDNA. Quantitative real-time PCR was conducted using a LightCycler FastStart DNA Master SYBR Green I kit (Takara, Dalian, China). The cycling conditions comprised 40 cycles of 95 °C for 15 s, 59 °C for 30 s, and 72 °C for 30 s using a single fluorescence measurement. Melting curve analysis, in which the temperature was increased from 59 °C to 95 °C at a heating rate of 0.2 °C/s using continuous fluorescence measurement, revealed a single, narrow peak of suspected fusion temperature. The mRNA levels of TNF-α, IL-23 and 1L-1β were calculated relative to amounts of a standard sample and normalized to the mRNA levels of 18 s. All reactions were performed using the StepOnePlus Real-Time PCR System (Applied Biosystems, Foster City, CA, USA).

### 4.10. Western Blot Analysis of NF-κB

To analyze the expression of NF-κB, BV-2 cells were lysed in a buffer containing 20 mM Tris–HCl (pH = 7.4), 1 mM EDTA, 140 mM NaCl, 1% (*w/v*) Nonidet P-40, 1 mM Na_3_VO_4_, 1 mM phenylmethylsulfonyl fluoride, 50 mM NaF, and 10 μg/mL aprotinin. The cell lysates were separated by 8% sodium dodecyl sulfate–polyacrylamide gel electrophoresis and electrotransferred onto polyvinylidene difluoride membranes (Bio-Rad, Hercules, CA, USA). The membranes were soaked in blocking buffer (1× Tris-buffered saline (TBS), 0.1% Tween 20, and 5% non-fat dry milk) for 1 h and incubated overnight at 4 °C with primary antibodies against NF-κB (Abcam, Cambridge, UK; 1:1000) and β-actin (Cell Signaling Technology, Danvers, MA, USA; 1:1000). The blots were developed using peroxidase-conjugated anti-mouse and anti-rabbit IgG and a chemiluminescent detection system (Santa Cruz Biotechnology, Dallas, Texas, USA). The bands were imaged using a ChemicDoc XRS system (Bio-Rad,) and analyzed using Quantity One imaging software (Bio-Rad). Each sample was assayed in triplicate and each experiment was performed twice.

### 4.11. Immunocytochemistry of NF-κB

After treatment with Aβ and/or LMDIR, BV-2 cells were fixed with 4% PFA for 20 min at room temperature. The cells were permeabilized with PBS containing 0.4% Triton X-100 for 20 min and were then blocked with TBS containing 10% normal goat serum and 0.02% Tween 20 for 1 h at room temperature. The cells were then incubated with a primary antibody against NF-κB (1:500, Abcam, Cambridge, UK) overnight at 4 °C in TBS containing 0.02% Tween 20 and 3% BSA. The BV-2 cells were then incubated with an Alexa Fluor 488-conjugated anti-rabbit IgG secondary antibody for 2 h at room temperature. NF-κB immunoreactivity was visualized using a Carl Zeiss LSM 700 Meta confocal microscope (Carl Zeiss AG, Oberkochen, Germany).

### 4.12. Statistical Analysis

All histological analyses were randomly performed with a blind manner for each group. All statistical analyses were performed using GraphPad Prism 7.0 software (GraphPad Software, Inc., La Jolla, CA, USA). Values were expressed as mean ± standard error of the mean (SEM). Paired samples statistics using the Student’s t-test was conducted for comparisons of 4G8-signals analysis between the sham-exposed and LMDIR-exposed groups. All other immunofluorescence analyses were performed using one-way analysis of variance followed by Fisher’s least significant difference post-hoc test for comparisons among the three groups. Differences with a *p*-value less than 0.05 were considered statistically significant.

## Supplementary Materials

Supplementary materials can be found at https://www.mdpi.com/1422-0067/21/10/3678/s1.

## Abbreviations

5XFAD	Five familial AD mutations
AD	Alzheimer’s disease
Aβ	Amyloid-β
DG	Dentate gyrus
DMEM	Dulbecco’s Modified Eagle Medium
DMSO	Dimethyl sulfoxide
GFAP	Glial fibrillary acidic protein
Gr	Granular layer
Iba-1	Ionized calcium-binding adaptor molecule 1
IL	Interleukin
LDIR	Low-dose ionizing radiation
LMDIR	Low–moderate dose ionizing radiation
ML	Molecular layer
MMP	Metalloproteinase
MTT	3-(4,5-dimethylthiazol-2-yl)-2,5-diphenyltetrazolium bromide
NeuN	Neuronal nuclei
NF-κB	Nuclear factor-kappa B
PB	Phosphate buffer
PBS	Phosphate-buffered saline
PDGF	Platelet-derived growth factor
Py	Pyramidal tract
ROI	Reactive oxygen intermediates
SLu	Stratum lucidum
SR	Stratum radiatum
Sub	Subiculum
SYN	Synaptophysin
TNF-α	Tumor necrosis factor-alpha
