# Supplementary material for: Neuroprotective and Anti-Inflammatory Effects of Low–Moderate Dose Ionizing Radiation in Models of Alzheimer’s Disease
Source: Int J Mol Sci. 2020 May 23;21(10):3678. doi: 10.3390/ijms21103678 (PMC7279400; doi:10.3390/ijms21103678)
Supplement: Supplementary file 1 [file ijms-21-03678-s001.docx]

**Supplementary Figures**

**
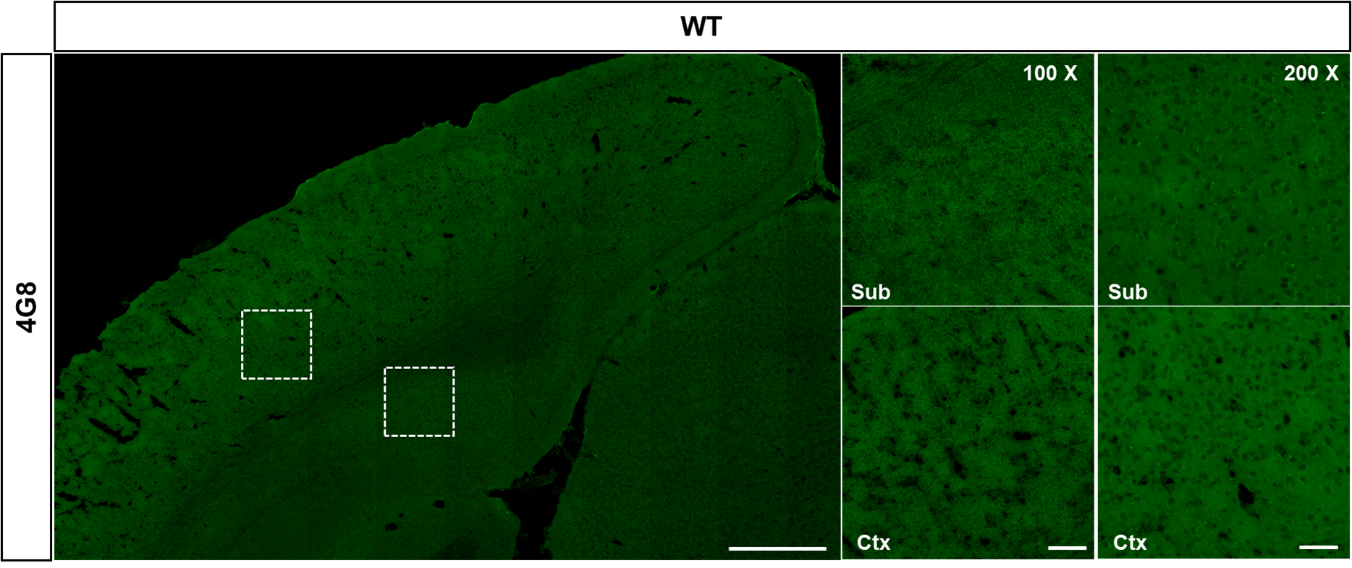
**

**Supplementary Figure 1.** Representative images of 4G8 immunoreactivity in the subiculum and cerebral cortex of sham-exposed wild-type mice. Scale bars denote 50 μm (100X), 100 μm (200X), and 500 μm (hemisphere). WT, wild-type; Sub, subiculum; Ctx, cerebral cortex.

**
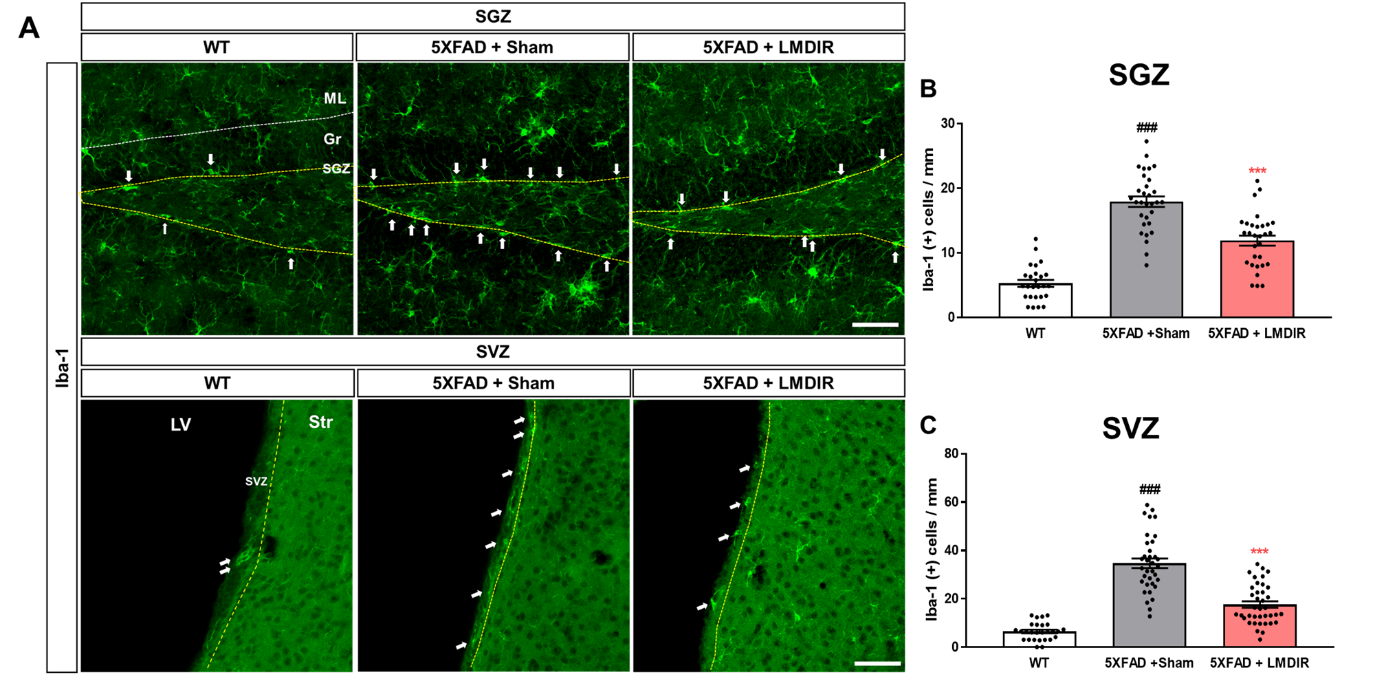
**

**Supplementary Figure 2.** Inhibitory effect of LMDIR on neuroinflammation in the subgranular zone (SGZ) and subventricular zone (SVZ) of 5XFAD mice. (A) Representative images of immunofluorescent staining of the SGZ and SVZ for Iba-1, a marker of microglia. (B-C) The number of Iba-1 (+) cells was significantly higher in the SGZ and SVZ of 5XFAD mice than that of the WT mice. In contrast, the number of Iba-1 (+) cells was significantly lower in the SGZ and SVZ of LMDIR-exposed 5XFAD mice than that of the sham-exposed 5XFAD mice. Data are presented as mean ± SEM (n = 7 in each group). ^###^ *p* < 0.001: WT mice versus sham-exposed 5XFAD mice. ^***^ *p* < 0.001: sham-exposed 5XFAD mice versus LMDIR-exposed 5XFAD mice. Scale bar= 40 μm. LMDIR, Low-moderate dose ionizing radiation; Iba-1, ionized calcium-binding adaptor molecule 1; SGZ, subgranular zone; SVZ, subventricular zone.

**
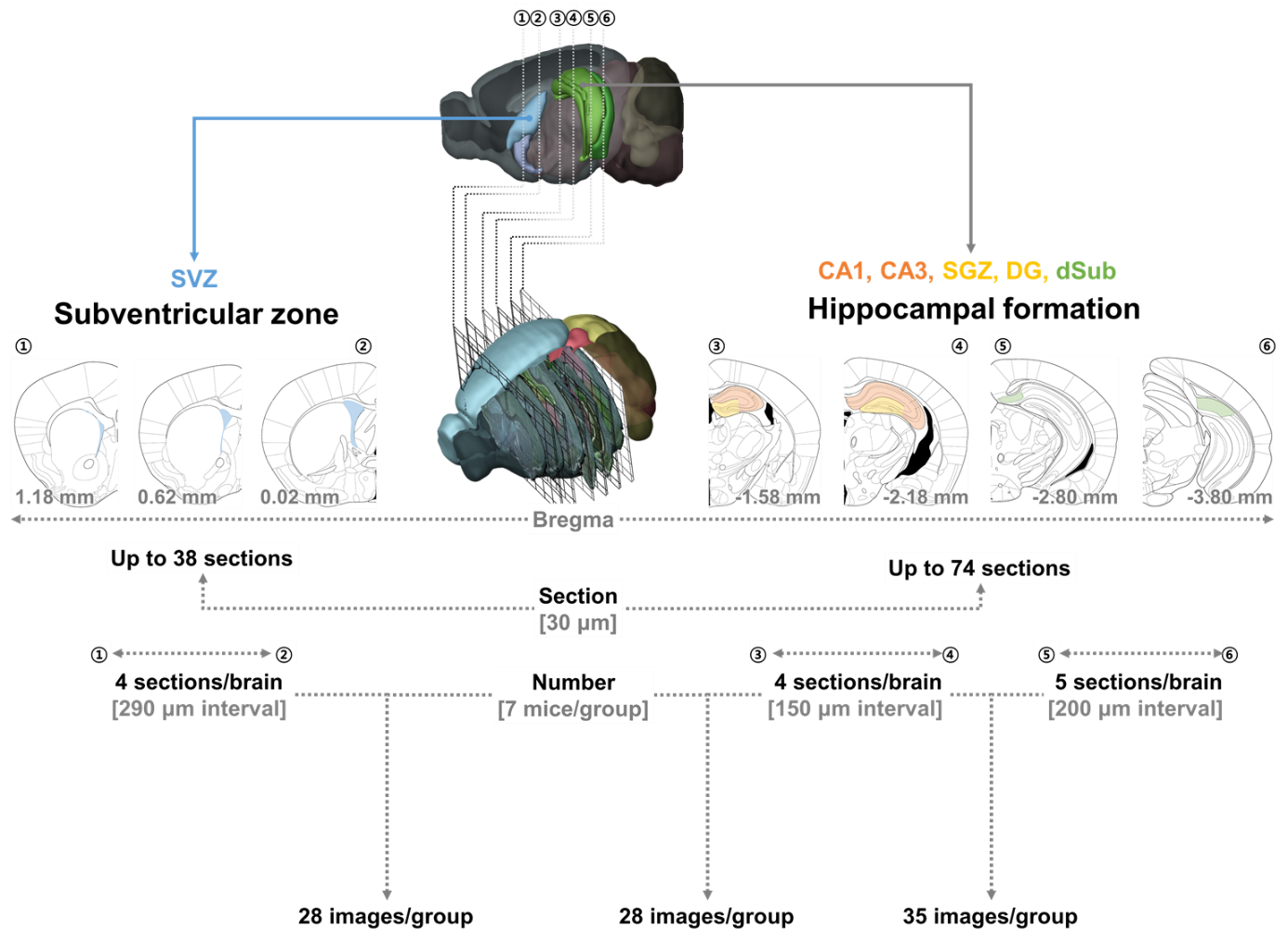
**

**Supplementary Figure 3.** Preparation of mice brain section for immunohistochemistry. From ① to ②, subventricular zone was designated from 1.18 mm to 0.2 mm from the bregma. From ③ to ④, hippocampal area was designated from -1.58 mm to -3.80 mm from the bregma. Fixed and cryoprotected mouse brains were coronally sectioned at a thickness of 30 μm in cryostat. Thirty-eight (subventricular zone) or seventy-four (hippocampal region) sections per mouse were obtained using the cryosection. In the subventricular zone, four sections per brain were taken from five mice at 290 μm intervals to obtain 28 images. In the hippocampal area, four to five sections per brain were taken from five mice at 150-200 μm intervals to obtain 28-35 images for CA1, CA3, DG, and dSub. The acquired images were subjected to topographical quantification and statistical analysis in a blind manner.
